# FINANCIAL OUTCOMES ASSOCIATED WITH COVID-19 IN NURSING HOMES

**DOI:** 10.1093/geroni/igad104.1144

**Published:** 2023-12-21

**Authors:** Gregory Orewa, Robert Weech-Maldonado, Sue Feldman, David Becker, Ganisher Davlyatov, Justin Lord

**Affiliations:** University of Texas, San Antonio, San Antonio, Texas, United States; University of Alabama at Birmingham, Birmingham, Alabama, United States; University of Alabama at Birmingham, Birmingham, Alabama, United States; University of Alabama at Birmingham, Birmingham, Alabama, United States; University of Oklahoma Health Sciences Center, Oklahoma City, Oklahoma, United States; Louisiana State University at Shreveport, Shreveport, Louisiana, United States

## Abstract

Financial stability and well-being are critical for the success of any organization. COVID-19 put nursing homes (NH) in a financial bind, affecting their costs and revenues and mounting pressure on them despite government intervention. NHs have been one of the weak links in our care continuum for the U.S. healthcare system, and the pandemic caused havoc in the industry. Given the pandemic impact on NH operating cost and revenue, this study examined the level to which financial performance in NHs deteriorated. Six different datasets were merged for 2018–2021: Medicare cost reports, Payroll-Based Journal, NH Compare, Area Health Resource File, HHS Provider Relief Fund, and CDC COVID-19 Data Tracker. The data was modeled using random-effects regression. Dependent variables were operating revenue per patient day, operating cost per patient day, and operating margin. Independent variable was pre- and post-COVID-19. Organizational, community-level, and COVID-19-related variables were used as controls. Results suggest that the impact of COVID-19 was associated with an increase in operating cost per patient day (β=56.64, p< 0.05), and a decrease in operating margin (β=-8.04, p< 0.05), but the associated operating revenue per patient day (β=26.78, p< 0.05), did not increase at the rate at which the operating cost increased. The increase in operating cost per patient day compared to the operating revenue per patient day was double. Given the negative impact of the COVID-19 pandemic on NH’s financial performance, policymakers should monitor the financial health of NH. Further deterioration in financial performance may result in NH closures and reduced access to long-term care.

